# Variability of defensin genes from a Mexican endemic Triatominae: *Triatoma (Meccus) pallidipennis* (Hemiptera: Reduviidae)

**DOI:** 10.1042/BSR20180988

**Published:** 2018-09-28

**Authors:** Paulina Díaz-Garrido, Omar Sepúlveda-Robles, Ignacio Martínez-Martínez, Bertha Espinoza

**Affiliations:** 1 Departamento de Inmunología, Instituto de Investigaciones Biomédicas, UNAM 04510, Ciudad de México, México; 2 Catedrático CONACyT – Unidad de Investigación Médica en Epidemiología Clínica UMAE-Hospital de Pediatría, Centro Médico Nacional Siglo XXI, Instituto Mexicano del Seguro Social, Ciudad de México

**Keywords:** antimicrobial peptides, Insect defensin, Triatoma pallidipennis

## Abstract

Chagas disease remains a serious health problem for countries where the most common mode of transmission is infection contracted from the feces of a Triatominae insect vector. In México, 32 species of Triatoma have been identified; amongst them, *Triatoma (Meccus) pallidipennis* is an endemic species reported to have high percentages of infection with *T. cruzi*. Defensins, cysteine-rich cationic peptides, are a family of antimicrobial peptides (AMPs); the synthesis of these molecules is crucial for insect’s immune defense. In the present study, the genes encoding defensins in *T. pallidipennis* were sequenced with the purpose of identifying the variability of these genes in a Mexican vector of *T. cruzi*. We found 12 different genes encoding three mature peptides, all of which had the typical folding of a functional insect defensin. In this work two Defensins type 1 and one type 4 were identified. The pro-peptide domain was highly variable and the mature peptide was not. This is the first report focus on variability of defensins from an epidemiologically important *Triatoma* in Mexico.

## Introduction

Chagas disease, one of the neglected tropical diseases, is caused by the protozoan *Trypanosoma cruzi*, the main transmission is through the feces of hematophagous insects of the Triatominae subfamily (Hemiptera: Reduviidae). It is estimated that this disease causes more than 10000 deaths per year and that 8 million people are infected with this parasite [1]. In México, this disease is spread by many species of *Triatoma*. Ramsey et al. (2015) [2] reported 31 different species in the country, including the endemic *Triatoma (Meccus) pallidipennis* (*T. pallidipennis*) that forms part of the Phyllosoma complex and colonizes extensive areas in the center of the country, living in domestic and peridomestic environments and more importantly with a natural infection index of 50% in some regions [3,4].

To control the parasite and pathogen proliferation, insect immune defense includes cellular and humoral responses. Humoral immunity consists of the generation of prophenoloxidase (PPO), hemolymph coagulation cascades, reactive oxygen species (ROS), reactive nitrogen species (RNS), and antimicrobial peptides (AMPs). Most AMPs are synthesized as inactive precursor proteins or propeptides, and active peptides or mature peptides are generated by proteolysis; these molecules have a broad spectrum of targetted organisms ranging from viruses to parasites and are produced by several species other than insects, including species of bacteria, plants, and vertebrates [5,6].

Most insect AMPs are small cationic/basic peptides with activities against bacteria and/or fungi, some AMPs also show activities against several parasites and viruses [7,8]. The fat body of insects is the main organ responsible for the production of AMPs, which can diffuse into the hemolymph and circulates throughout the entire insect body [9]. They can also be secreted by the gut epithelial cells, where parasites such as *T. cruzi* might interact directly with them, in addition to other potential pathogens acquired during feeding [10,11].

Insect defensins are a family of AMPs, distinguished for having six conserved cysteine residues that are linked in three pairs of disulphide bridges (Cys^I^–Cys^IV^, Cys^II^–Cys^V^, and Cys^III^–Cys^VI^) and form a consensus folding pattern consisting of the cysteine-stabilized α-helix β-sheet (CSαβ) motif that is the active site of the domain for antimicrobial activity [12–16]. These cationic peptides have a molecular mass of approximately 4 kDa, are typically 33–46 amino acid residues long and show activity mainly against Gram-positive bacteria through a channel-forming mechanism [5,17,18].

Several AMPs have already been found in different species of Triatominae such as *Rhodnius prolixus, T. brasiliensis* and *T. infestans*. The only sequenced genome of a hemipteran is that of *R. prolixus*, where 11 genes of defensins were identified [19]. Two transcriptomic analyses of *T. pallidipennis* have been reported, including one of the salivary glands, where no AMPs were found [20]. The other transcriptomic analysis reported eight transcripts of defensins and three transcripts of lysozymes [21].

These facts and their importance for immune response are reasons to explore in detail the variability of the defensin-encoding genes in other important Triatominae vectors of Chagas disease.

Most of the published studies on this topic focus on the expression of mRNA of South American species such as *T. infestans* and some members of the *T. brasiliensis* complex *(T. brasiliensis, T. sherlocki*, or *T. juazeirensis*), where the expression of these molecules in different organs has been analyzed [22–26].

Information on other important vectors of Chagas disease from the northern part of the continent is missing, for this reason the aim of the present study was to analyze the variability of defensin genes in the genome of the Mexican endemic bug *T. pallidipennis*. We present a comparative analysis with other defensins previously described and the predicted structures of the mature peptides using two different computational tools for the modeling of the molecules. The presented results offer the possibility of interpreting the phylogenetic relationships of hemipteran defensins, and the predicted mature peptide sequences could provide information for the development of novel drug targets.

## Materials and methods

### Insect origin and maintenance

For this work, we used adult females of *T. pallidipennis*. The insects were maintained in a colony at the Laboratorio de Estudios sobre la Tripanosomiasis Americana, Instituto de Investigaciones Biomédicas, UNAM, under controlled temperature and humidity. The insects were fed with rabbit blood, and all the procedures were carried out according to the Ethic Code of the Instituto de Investigaciones Biomédicas for animal care.

### Genomic DNA isolation

Genomic DNA was isolated from both the fat body and digestive tract (washed several times with saline solution to eliminate intestinal contents) and combined; the organs were macerated in 1 ml of lysis solution (50 mM Tris/HCl, pH 8; 100 mM EDTA, pH 8; 100 mM NaCl; 1% SDS, and 20 µg/ml RNase) and incubated at 37°C overnight. The phenol chloroform technique was used to extract DNA [27]. Nucleic acid integrity and concentration were quantitated using a NanoDrop 1000 (Thermo Scientific) and checked by gel electrophoresis. The DNA was kept at 4°C until used.

### Gene amplification cloning and sequencing

The specific forward and reverse primers used to amplify defensin genes of *T. pallidipennis* were designed using the sequences encoding Def1, Def3, and Def4 of *T. brasiliensis* reported by Araújo et al. (2006) [22] and Waniek et al. (2009) [25]: [Def1 – Fwd (5′-GGCGCCATGAAGTGCGCACTCTCTTTG-3′), Def1 – Rev (5′-CATTGCAGGAAGTGAATTGATGCTTGGG-3′), Def3 – Fwd (5′-GGCGCCATGAAGTGTGCACTCTCTTTG-3′), Def3 – Rev (5′-CTATCTGTCACTGCAGGAAGTGAGCTTGGG-3′), Def4 – Fwd (5′-GGCGCCATCATGAAGTGTGCACTCTC-3′) and Def4 – Rev (5′-CACTGCAGGAAGTGAAGAGTGCTTGGG-3′)]. PCRs were performed in a T100 thermal cycler (Bio-Rad, Inc); after one initial cycle of 95°C for 5 min, PCR was carried out for 30 cycles (1 min at 94°C, 1 min of Tm at 66°C (Def1-oligos) or 70°C (Def3/Def4-oligos), and 1 min at 72°C), followed by a final elongation step of 10 min at 72°C. The putative defensin genes that were amplified (~400 bp) were purified by cutting the gel band and cloning into pJET using a CloneJet PCR Cloning Kit (Thermo Fisher Scientific, Inc) following the manufacturer’s instructions. Finally, plasmids were isolated from 15 randomly selected clones and sequenced (five sequences per gene: Def1, Def3, and Def4) using a pJET1.2 forward sequencing primer (5′-CGACTCACTATAGGGAGAGCGGC-3′). The sequencing was done with the Sanger technique, using ABI PRISM 3100 equipment.

### Analysis of sequences and identities

*T. pallidipennis* intron and mRNA sequence identification was performed by alignment using the data from the GenBank Def1 (AY641574.1), Def3 (EU694177.1), and Def4 (EU694178.1) mRNA nucleotide sequences of *T. brasiliensis*. The amino acid sequences were deduced with the translate tool from ExPASy and aligned with the MultAlin server http://multalin.toulouse.inra.fr/multalin/multalin.html [28]. Predicted signal peptide cleavage sites and propeptide cleavage sites were calculated using the ProP 1.0 Server of Technical University of Denmark (http://www.cbs.dtu.dk/services/ProP/). Molecular masses, isoelectric points, and other protein parameters were determined with the ExPASy Bioinformatics Resource Portal web server.

### Phylogenetic analysis

Molecular phylogenetic analyses were conducted with the maximum likelihood (ML) method in MEGA6 software, based on the Tamura 3-parameter model. A discrete γ distribution was used to model evolutionary rate differences amongst sites (five categories (+G, parameter = 2.2566)). The rate variation model allowed some sites to be evolutionarily invariable. The best model was established from a set of 465 nts. There was a total of 465 positions in the final dataset. The analysis involved 32 nucleotide sequences. The codon positions included were 1st+2nd+3rd+noncoding. Ten thousand replicates were used for the analyses. Bayesian inferences (BIs) of *T. pallidipennis* defensin relationships were performed using Mrbayes software. Support node values were obtained from 1000000 generations and are shown next to the branches. The evolutionary distances were computed using the GTR substitution model with γ-distributed rate variation across sites and a proportion of invariable sites. The analysis involved a final dataset of 32 nucleotide sequences and a total of 465 positions (gaps included). The analysis was terminated when the S.D. of split frequencies = 0.0091. A matrix of genetic distances was calculated with MEGA6 software with the data used for the ML analysis.

### Structure prediction of mature peptides

The three sequences coding for the mature peptides were sent to the I-TASSER server for tertiary structure prediction [29–31], which is available at http://zhanglab.ccmb.med.umich.edu/I-TASSER/. The models were refined by energy minimization using YASARA (15097).

## Results

### Characteristics of the defensin genes from *T. pallidipennis*

Oligonucleotides that comprised the 5′ and 3′ UTR regions were generated using the previously reported sequences of *T. brasiliensis* Def1, Def3, and Def4. Using genomic DNA as a template and PCR amplification, we obtained amplicons of 400 bp for each gene. The PCR products were cloned into the pJET plasmid to obtain complete gene sequences.

**Figure 1:**
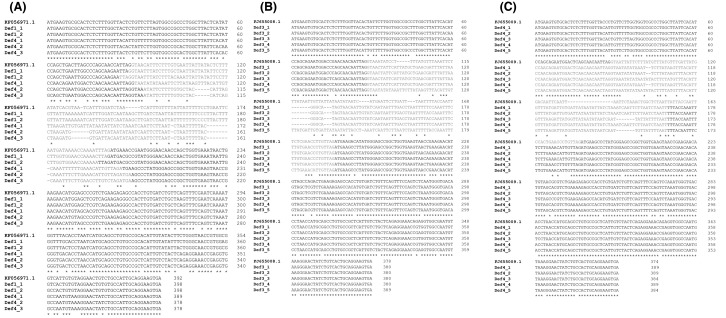
Defensins gene sequences of *Triatoma (Meccus) pallidipennis* Each alignment consists of five sequences per gen: Def1, Def3, and Def4. The sequences consist of two exons and one intron. Each set of sequences was aligned with Def1 (KF056971.1**)**, Def3 (FJ655008.1), and Def4 (FJ655009.1) from *T. brasiliensis*. (**A**) The set from Def1, varies both in size and position and all the sequences are different. (**B**) From Def3, the position of the intron is the same for all the sequences, also Def3.1 and Def3.4 are identical. (**C**) In the Def4 set, the position of the intron is the same, Def4.1, Def4.2, and Def4.4 are identical. The intron is presented in gray color. Identical nucleotides between the sequences is indicated by asterisks.

The 15 sequences of *T. pallidipennis* defensins obtained (five for each gene) were aligned with the defensin sequences reported for *T. brasiliensis* to identify the intron position (Figure 1). From these 15 sequences obtained initially, 12 different genes were identified (GenBank accession numbers: MH000324, MH000325, MH000326, MH000327, MH000328, MH000329, MH000330, MH000331, MH000332, MH000333, MH000334, and MH000335).

The sequences obtained with the Def1 oligos present more variability in both the position and the size of the intron as well as in its composition. The introns begin between positions 88 and 93, while the sizes of the introns oscillate between 95 and 112 bp (Figure 1A). The sequences obtained from the amplicons generated with the Def3 oligos are more homogeneous; 3.1 and 3.4 have the same sequence, and in all the sequences, the intron begins at position 89 and ends at position 186. Therefore, the sequences have a size of 97 bp. However, Def3.5 has 106 bp, since it ends at position 195 (Figure 1B). Finally, the sequences obtained with the Def4 oligos, Def4.1, Def4.2, and Def4.4, are the same; the intron begins at position 61 and ends at nucleotide 167, for a length of 106 bp. In the case of Def4.3 and Def4.5, the intron ends at position 162 and consists of 101 bp (Figure 1C).

### Characterization of amino acids of defensin genes from *T. pallidipennis*

**Figure 2:**
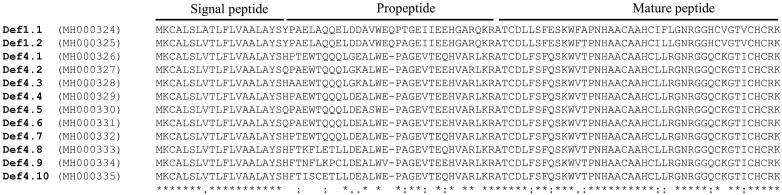
Amino acids translated from gene-coding defensins The 12 different sequences were translated to amino acids, only two correspond to defensin type 1 (Def1.1 and Def1.2), which have an extra aa (Q). The others ten sequences, correspond to defensin type4, in which the propeptide is the more variable region. Only three different mature peptides were obtained. Identical amino acid residues between the sequences are indicated below the alignment sequences by *; (:) indicates conservation between groups of strongly similar properties and (.) indicates conservation between groups of weakly similar properties. The signal peptide, propeptide, and mature peptide are marked.

**Figure 3:**
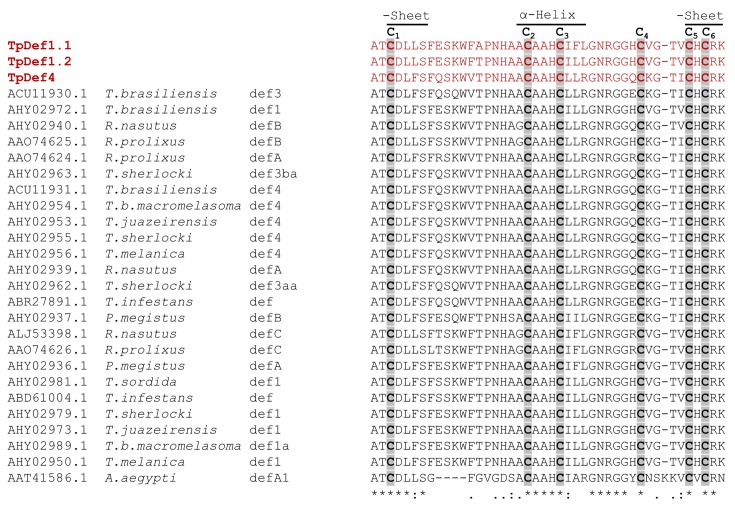
Aligment of predicted *T. pallidipennis* defensins with other Triatominae defensins Twenty-five defensins’ sequences were alignment with TpDef1.1, TpDef1.2, and TpDef4. All the sequences showed the six cysteines in the same position and the sequences have the same size. TpDefs from *T. pallidipennis* are in red bold. Identical and similar amino acid residues between the defensins are indicated below the alignment sequences by asterisks and dots respectively; (:) indicate conservative substitution, (.) indicate less consevative substitution. Cysteine residues forming the disulphide bridges are gray shaded. The sequence of *A. aegypti* is shown for comparison.

The genes were edited *in situ*, and bioinformatics analysis was performed to identify the signal peptide, propeptide, and mature peptide (Figure 2). Of the 12 sequences, 10 had 93 aa, and two had 94 aa, with those in the latter category having an extra Glutamine (Q) at position 36 (Def1.1 and Def1.2). The signal peptide is the same for almost all the sequences [MKCALSLVTLFLVAALAYS], however Def1.1 have Alanina (A) on the 8th position unlike the others sequences that have Valine (V) in this position; nevertheless, the propeptide is the most variable region, since eight different sequences were obtained, and all of them present the cleavage site KR (Lysine-Arginine). Interestingly, from the 12 different sequences, only 3 different mature peptides were found. Two correspond to Def1 (named TpDef1.1 and TpDef1.2), with only one different aa Alanine (A) for Threonine (T). The other ten sequences coded for the same mature *T. pallidipennis* defensin, which was named TpDef4 (Figure 3). An alignment was made using the 3 different mature peptides and 24 reported sequences of defensins in other *Triatoma* species, including one defensin from *Aedes aegypti*. All the sequences have six characteristic cysteines in the same position; furthermore, the size is the same for all the sequences, except for the dipteran defensin.

### Phylogenetic analysis

**Figure 4:**
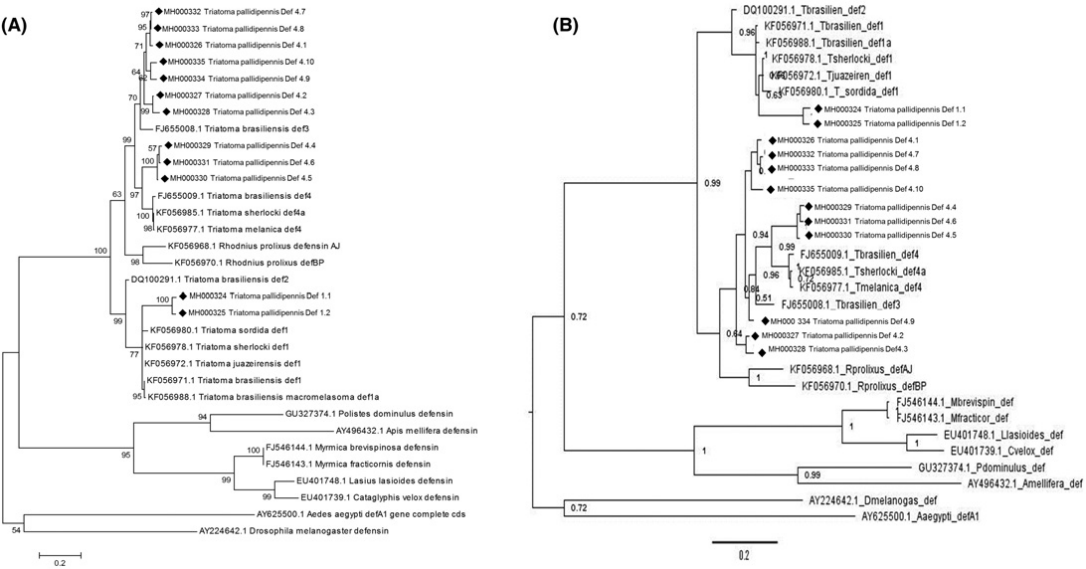
Phylogenetic analysis of defensins (**A**) Molecular phylogenetic analysis by ML method**.** The evolutionary history was inferred by using the ML method based on the Tamura 3-parameter model. Initial tree(s) for the heuristic search were obtained by applying the Neighbor-Joining method to a matrix of pairwise distances estimated using the Maximum Composite Likelihood (MCL) approach. A discrete γ distribution was used to model evolutionary rate differences amongst sites. The rate variation model allowed for some sites to be evolutionarily invariable. There were a total of 465 positions in the final dataset. Evolutionary analyses were conducted in MEGA6. (**B**) Bayesian analysis of *T. pallidipennis* defensin. Phylogenetic relationship was stablished using the Mrbayes software. The node values were obtained from 1000000 generations and were showed next to the branches. The evolutionary distances were computed using the GTR substitution model with γ-distributed rate variation across sites and a proportion of invariable sites. The analysis involved 32 nucleotide sequences and a total of 465 positions in the final dataset (gaps including). S.D. deviation of split frequencies = 0.0091. ♦ *T. pallidipennis* defensins.

ML and BI phylogenetic analyses were performed to establish the phylogeny of defensin sequences of *T. pallidipennis* with respect to other sequences of South American Triatominae. The trees from the BI and ML analyses were nearly identical in topology, since all the Def1 sequences are grouped in the same clade, and the Def4 sequences are grouped in a different clade that shows subclades and branch lengths at only a few nodes. Both methods showed Triatominae defensins as a monophyletic group with high support. In the gene tree inferred from ML and BI analyses, the TpDef1.1 and TpDef1.2 sequences are situated in the Triatominae defensin 1 clade with strong statistical support. However, in both analyses, defensin 1 sequences of *T. pallidipennis* show clear differences compared with the defensin 1 sequences of the South American Triatominae species, which form a subgroup with strong support. Additionally, the length of the branch shows that defensin 1 sequences of *T. pallidipennis* have accumulated a greater number of changes than the other sequences of the same subgroup (Figure 4).

In contrast, the sequences of defensins 4.1 to 4.10 show diversity. Some of them are more similar to the defensin 4 sequences reported in South American Triatominae species, while others form clearly separated subgroups. However, amongst them, there is similarity, which is evident when comparing their genetic distances (Table 1).

**Table 1:** Genetic distances between defensins

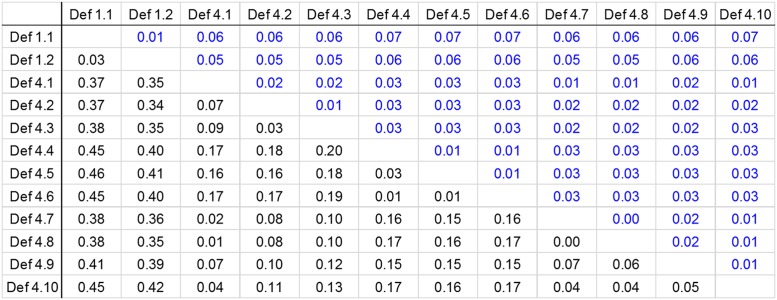

### Modeling by homology

**Figure 5:**
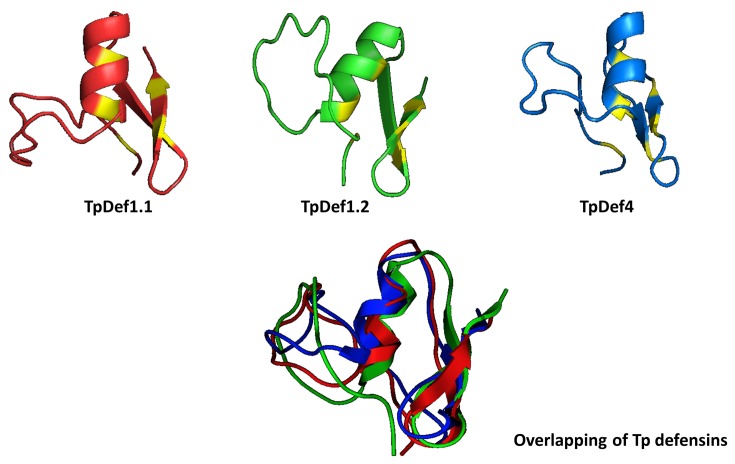
Molecular modeling of Tp defensins Theoric mature peptides were generated using I-TASSER. All exhibit the same structural topology conformed by three disulphide bonds as well as an α-helix and β-sheets, which are known as cysteine-stabilized (CSαβ) motif. Cysteines are marked in yellow. An overlapping of the three mature peptides is also shown.

Molecular modeling of the three theoretical mature peptides was carried out using I-TASSER. All the mature peptides exhibit the same structural topology characterized by three disulphide bonds, an α-helix and β-sheets, which are known as the cysteine-stabilized (CSαβ) motif. TpDef1.1 and TpDef1.2 differ only in two amino acid residues: Alanine replaced by Tryptophan (W), localized in the loop, and Phenylalanine (F) replaced by Leucine (L) in the helix. The TpDef4 sequences have eight different amino acids with respect to TpDef1.1 and TpDef1.2; these changes are located throughout the secondary structure. When overlapping the models of these peptides, it is obvious that the changes in the amino acids do not alter the final conformation of the molecules (Figure 5).

## Discussion

A high degree of sequence conservation exists in invertebrate defensins [32,33], which indicates the biological importance of these molecules. In Hemipteran insects, previous studies have demonstrated that the expression of these peptides not only varies in the organ/tissue of origin but also depends on the type of pathogen to which the insect is exposed [34,35].

The immune system in the midgut of insects is one of the most exposed tissues since it is always in contact with a variety of microorganisms. *T. cruzi* is only found in the digestive tract of the Triatominae, so the production of AMPs in the insect gut is therefore vital to protect against infection and to maintain homeostasis of the intestinal microbiota [36,37]. Yet, few studies focus on the expression of the defensin-encoding genes in the presence of *T. cruzi.* One study in which *R. prolixus* was infected with different *T. cruzi* strains showed that the infection with Dm 28c induced an increase in defensin transcripts in contrast with the infection with Y strain [11,37]. It suggests that depending on the genetic linage of the *T. cruzi* strain the defensing response could be different.

With respect to the percentage of infection by *T. cruzi* and defensin expression there are few studies, nevertheless, *T. pallidipennis* seems to have less infection than *T. dimidiata* or *T. barberi* [3,38], indicating a possible role in the diversity of defensins on the control of parasitemia. But more studies need to be done to clearly elucidate this point.

In the present study female insects were used. It will be interesting to analyze male insects in the future, but until now there are few studies that focus in the parasitemia related to the sex of the insects and they do not show a clear correlation between sex and infection mainly because the number of insects was small and difference in the number of females and males infected was not significant [3,39]. In our personal experience both females and males have similar rates of infections (data not published).

We characterized 12 genes that code for defensins in a Mexican endemic *Triatoma*, and our results indicate that the variability of the genes lies mainly in the introns (position, composition, and size). The 12 ORFs presented a signal peptide with 19 amino acid residues, and the largest difference amongst the amino acid sequences is at the propeptide level. The sequences Def1.1 and Def1.2 have 32 amino acid residues in the propeptide region, while the other sequences have 31 amino acids. Additionally, the KR cleavage site at the end of the propeptide region is conserved in all sequences and in other Triatominae defensins [34].

Three mature peptides from the 12 sequenced genes were obtained, named TpDef1.1, TpDef1.2, and TpDef4. Furthermore, the intermediate region in the signal peptide and the mature peptide is highly variable, as has been reported for *R. prolixus* [34]. The specific biological function of the intermediate region is unknown, but it can participate in the cellular trafficking of the immature peptide; this has been reported for the human BMP protein, for which changes in the sequence of this region slow the secretion of this peptide [40].

The results indicate that the mature peptide is highly conserved and similar to all Hemipteran sequences used in the comparative analysis, especially at the position of the six cysteine residues that permit the formation of three disulphide bridges. These data differ from those reported for mammalian defensins, particularly with β-defensins, where a high variability in the antimicrobial domain has been identified and the functional diversity may depend on the structural variation in the β-defensins. This may be due to the diverse and changing microbial environment in their habitats and for a better deal with of a broader range of pathogens [41].

In the phylogenetic analyses using the genomic sequences, a clear separation of the Triatominae defensins from the sequences of other arthropods was observed, as previously reported [42]. In the Triatominae branch of the phylogenetic trees, a clear separation of two groups of defensins was observed: two type-1 sequences and ten type-4 sequences. The type-1 defensin sequences of *T. pallidipennis* are clearly distant from the South American species sequences, a result of a greater accumulation of changes, reflected in a greater length of branches in the phylogenetic tree (Figure 4). Something similar has been observed when comparing other *T. pallidipennis* proteins such as triafestins and triabins, which, despite their similarity in function and structure, are clearly separated from their South American counterparts [43]. However, despite these differences, Def 1.1 and Def 1.2 of *T. pallidipennis* show a close evolutionary relationship as members of the defensin 1 clade. The sequences of defensin type 4 of *T. pallidipennis* are grouped in the phylogenetic trees and separated from the type-1 defensin sequences.

Additionally, the defensins of the *Triatoma* genus are clearly separated from the *Rhodnius* genus sequences, as previously described [26]. Furthermore, three sequences (Def 4.4, Def 4.5, and Def 4.6) in both the ML and Bayesian analyses are closely related to type-4 defensins of *T. brasiliensis, T. sherlocki*, and *T. melanica*, suggesting a close evolutionary relationship amongst them. TpDef4 is coded by ten sequences (Def4.1–Def4.10). This result suggests that this defensin has experienced a duplication phenomenon for retroposition or nonallelic homologous recombination between transposable elements [44], indicating their possible relevance, as has been demonstrated with A and J defensins that are secreted in the intestine and fat body in *R. prolixus* in response to bacterial challenge [34]. This phenomenon should be explored further, since it has been shown that some families of human defensins such as DEFB4 have between 2 and 12 copies per genome [45]. Another possibility is that each of these sequences is regulated by different elements in the flanking 5′ region, as has been demonstrated for other sequences of AMPs in arthropods such as *Manduca sexta* [46].

Computational modeling shows that, although the three mature peptides have some different amino acids, this does not affect the final folding of the peptide. All the mature peptides presented three disulphide bridges formed by six cysteine residues and three characteristic domains: an N-terminal flexible loop, followed by an α-helix and two C-terminal antiparallel β-sheets.

Recently, using transcriptome analysis, researchers have reported that *T. pallidipennis* expressed mRNA for eight defensin genes. One of these corresponds to Def4.6; other, to Def1.2 [21], the dataset is available at http://201.131.57.23:8080/data/triatoma. That report is in accordance with our finding of divergence in defensin expression on *T. pallidipennis.*

Many questions remain to be answered, such as the following: are the peptides reported here functional? Which are the stimuli that induce the secretion of these peptides? Which organs secreted them? What is the meaning of the sequence diversity in the intron region, if there is any? Furthermore, we are currently analyzing the expression of defensin in infected *T. pallidipennis* and in different stages of the insect.

The present work initiated the genetic characterization of this molecule in an important North American vector of Chagas disease, a human malady that causes thousands of deaths. Knowledge about these molecules can help in designing novel strategies for the control of Chagas disease vectors.

## Abbreviations

AMP: antimicrobial peptide
BI: Bayesian inference
CSαβ: cysteine-stabilized α-helix β-sheet
ML: maximum likelihood
