# Correction: Variability of defensin genes from a Mexican endemic Triatominae: Triatoma (Meccus) pallidipennis (Hemiptera: Reduviidae)

**DOI:** 10.1042/BSR-20180988_COR

**Published:** 2020-06-22

**Keywords:** Antimicrobial peptides, Insect defensin, Triatoma pallidipennis

The authors of the original article “Variability of defensin genes from a Mexican endemic Triatominae: *Triatoma (Meccus) pallidipennis* (Hemiptera: Reduviidae)” (*Bioscience Reports* (2018) **38**, https://doi.org/10.1042/BSR20180988) would like to add the following Acknowledgements to their study:

